# Supplementary material for: Toll-like Receptor 3 in the Hybrid Yellow Catfish (Pelteobagrus fulvidraco ♀ × P. vachelli ♂): Protein Structure, Evolution and Immune Response to Exogenous Aeromonas hydrophila and Poly (I:C) Stimuli
Source: Animals (Basel). 2023 Jan 14;13(2):288. doi: 10.3390/ani13020288 (PMC9854889; doi:10.3390/ani13020288)
Supplement: Supplementary file 1 [file animals-13-00288-s001.zip › Table S1.pdf]

animals MDPI

Table S1. NCBI accession numbers of protein sequences used in this study.

Species name	Accession number
Bactrocera dorsalis	XP 011205769.1
Galleria mellonella	XP 026749126.1
Bicyclus anynana	XP 023950341.1
Photinus pyralis	XP 031355790.1
Penaeus japonicus	AUG89725.1
Penaeus monodon	AYF59256.1
Myzus persicae	XP 022180533.1
Bactrocera tryoni	XP 039957006.1
Tribolium castaneum	XP 008195157.1
Ceratina calcarata	XP 017884489.2
Clupea harengus	XP 031415824.1
Chanos chanos	XP 030643535.1
Ctenopharyngodon idella	ABI64155.1
Triplophysa tibetana	KAA0722437.1
Ictalurus punctatus	ABD93873.1
Tachysurus fulvidraco	XP 026990765.1
Bagarius yarrelli	TSN95706.1
Oncorhynchus kisutch	XP 020352764.2
Gadus morhua	XP 030207631.1
Takifugu rubripes	XP 003972357.2
Oreochromis aureus	XP 031585665.1
Echeneis naucrates	XP 029361177.1
Scophthalmus maximus	AHW76803.1
Lateolabrax maculatus	QDE10493.1
Siniperca chuatsi	QEU52192.1
Collichthys lucidus	TKS68449.1
Sparus aurata	XP 030289726.1
Callorhinchus milii	XP 007891010.2
Amblyraja radiata	XP 032874464.1
Rhincodon typus	XP 020375140.1
Chiloscyllium griseum	AHG94983.1
Xenopus tropicalis	XP 002934448.3
Rana temporaria	XP 040190280.1
Bufo bufo	XP 040274690.1
Rhinatrema bivittatum	XP 029442799.1
Microcaecilia unicolor	XP 030047344.1
Echinops telfairi	XP 004696530.1
Mus pahari	XP 029387822.1
Mus musculus	AAK26117.1
Nomascus leucogenys	XP 003271546.1
Lynx pardinus	VFV22925.1
Suricata suricatta	XP 029783259.1
Camelus dromedarius	KAB1257730.1
Vicugna pacos	XP 015104650.1
Delphinapterus leucas	XP 030618416.1
Ovis aries	CAQ37823.1

Bubalus bubalis	ADY18594.1
Phasianus colchicus	XP 031464673.1
Anas platyrhynchos	QFG75926.1
Aquila chrysaetos chrysaetos	XP 029881255.1
Serinus canaria	XP 009099742.1
Geospiza fortis	XP 005415573.1
Camarhynchus parvulus	XP 030803195.1
Pelodiscus sinensis	XP 006128521.1
Chelonia mydas	XP 007063701.1
Terrapene carolina triunguis	XP 024075011.2
Mauremys reevesii	XP 039398304.1
Gopherus evgoodei	XP 030420380.1
Chelonoidis abingdonii	XP 032655079.1
