# Supplementary material for: Toll-like Receptor 3 in the Hybrid Yellow Catfish (Pelteobagrus fulvidraco ♀ × P. vachelli ♂): Protein Structure, Evolution and Immune Response to Exogenous Aeromonas hydrophila and Poly (I:C) Stimuli
Source: Animals (Basel). 2023 Jan 14;13(2):288. doi: 10.3390/ani13020288 (PMC9854889; doi:10.3390/ani13020288)
Supplement: Supplementary file 1 [file animals-13-00288-s001.zip › Table S2.pdf]

Table S2. Detailed sequence descriptions of the 10 TLR3 genes.

Species	Nucleotide Accession Number	Full length (bp)	ORF (bp)	5'-UTR (bp)	3'-UTR (bp)	Protein Accession Number	Predicted protein (aa)	Molecular Weight (kDa)	Theoretical pI	Signal peptide	Transmembrane
Pelteobagrus fulvidraco	XM_027134964.1	3553	2718	480	355	XP_026990765.1	905	103.82	8.46	YES	YES
Acanthochromis polyacanthus	XM_022211610.1	3359	2739	438	182	XP_022067302.1	912	103.73	8.83	YES	YES
Argyrosomus japonicus	MT956601	2739	2739	0	0	QOS44501.1	912	102.65	8.61	YES	YES
Cheilinus undulatus	XM_041786405	3218	2742	270	206	XP_041642339.1	913	102.82	9.18	YES	YES
Dicentrarchus labrax	FQ310508.3	2871	2871	0	0	CBN82176.1	957	110.24	8.93	NO	YES
Ictalurus punctatus	DQ423776	3319	2715	306	298	ABD93873.1	905	103.64	8.44	YES	YES
Megalops cyprinoides	XM_036516980	2754	2709	42	3	XP_036372873.1	903	102.64	8.89	YES	YES
Oreochromis niloticus	XM_025908386	3197	2736	191	270	XP_025764171.1	912	102.89	8.62	YES	YES
Salmo trutta	XM_029749902	5578	2739	304	2535	XP_029605762.1	913	102.16	8.23	YES	YES
